# Urology Coverage for Obstetric and Gynecological Operative Procedures Should Go Hand in Hand in a Hospital Setting

**DOI:** 10.1055/s-0041-1739165

**Published:** 2021-12-06

**Authors:** Tariq Faisal Al-Shaiji

**Affiliations:** 1 Urology Unit (Head), Department of Surgery, Jaber Al-Ahmad Al-Jaber Al-Sabah Hospital, Kuwait City, Kuwait

Dear Editor,

Iatrogenic operative injuries sustained to the urinary tract are not uncommon during obstetric and gynecological (Obs&Gyn) surgery. The ureters and bladder are usually involved. These injuries are among the most serious complications of Obs&Gyn surgery. About 75% of iatrogenic injuries to the urinary tract are due to Obs&Gyn surgical interventions, whereas injuries to the urinary tract complicate approximately 1% of all Obs&Gyn procedures 1 2 3 due to improper recognition and management. delayed diagnosis may lead to high morbidity or mortality. 3 Therefore, awareness and proper expert management of these injuries are of paramount importance. A study by Hammad et al. 4 demonstrated that the presence of instrumentation related to endourology in the Obs&Gyn operating theatre assisted the recognition and treatment of injuries to the urological system.

There has been a rise in what is known as exclusive maternity hospitals offering comprehensive but Obs&Gyn services. These hospitals or specialized centers tend to offer services pertaining to obstetrics and gynecology conditions only. They do not cover any other medical or surgical services. In this case, there are some possible scenarios.

1. The operating obstetrician or gynecologist has experience in urological surgery and would deal with it. This seems to be an uncommon situation. Komatsu et al. 5 examined through a questionnaire the opinions of Japanese obstetricians and gynecologists regarding training in other departments. Among 120 respondents, only 5 physicians (4.2%) revealed having experience in urology. A total of 90% stated that knowledge and skills in urology were required. In addition, 49.2% stated they would feel under stress if going to undertake training in other departments because of the inability to secure enough cases or the shortage of personnel.
2. Asking for assistance from an in-house general surgeon, who, if present, may not have the adequate urological experience.
3. Calling an adjacent hospital with Urology coverage to come to the rescue, with a possible delay in response, as well as the possibility that the ‘adjacent’ hospital is not be too ‘adjacent’.
4. Asking a favor from a colleague Urologist to intervene even when he or she is not on duty.

Indeed, this entire process would have a negative impact on the patients and health care providers. I have had close contact with both Urology and Obs&Gyn specialists locally, regionally, and internationally, who clearly had valid concerns regarding these stand-alone hospitals. One obvious concern from the point of view of the Obs&Gyn specialist is the lack of urgent support should such an injury occur. On the other hand, one concern from the perspective of Urology specialists is having too short of a notice to provide urgent assistance in the operating room at a hospital not covered under his or her premises (areas not covered geographically when on call for Urology) or during a time when he or she are being off duty. The aforementioned options are not ideal, and call for better planning, so that such specialized hospitals or centers have 24/7 Urology coverage preferably a supporting in-house unit or supporting individuals with expertise. This should also be the case in any resource-limited hospital. By doing so, both patients and physicians would be better served.
